# Fever Time Series Analysis Using Slope Entropy. Application to Early Unobtrusive Differential Diagnosis

**DOI:** 10.3390/e22091034

**Published:** 2020-09-15

**Authors:** David Cuesta-Frau, Pradeepa H. Dakappa, Chakrapani Mahabala, Arjun R. Gupta

**Affiliations:** 1 Technological Institute of Informatics, Universitat Politècnica de València, Alcoi Campus, 03801 Alcoi, Spain; 2 Clinical Pharmacology, Nanjappa Hospitals, Shimoga 91903, India; pradeepahd@nanjappahospitals.com; 3 Department of Medicine, Kasturba Medical College, Mangalore, Manipal Academy of Higher Education, Manipal 575001, India; chakrapani.m@manipal.edu (C.M.); arjunr333@gmail.com (A.R.G.)

**Keywords:** Slope Entropy, time series classification, body temperature, fever, Matthews Correlation Coefficient, malaria, dengue, differential diagnosis

## Abstract

Fever is a readily measurable physiological response that has been used in medicine for centuries. However, the information provided has been greatly limited by a plain thresholding approach, overlooking the additional information provided by temporal variations and temperature values below such threshold that are also representative of the subject status. In this paper, we propose to utilize continuous body temperature time series of patients that developed a fever, in order to apply a method capable of diagnosing the specific underlying fever cause only by means of a pattern relative frequency analysis. This analysis was based on a recently proposed measure, Slope Entropy, applied to a variety of records coming from dengue and malaria patients, among other fever diseases. After an input parameter customization, a classification analysis of malaria and dengue records took place, quantified by the Matthews Correlation Coefficient. This classification yielded a high accuracy, with more than 90% of the records correctly labelled in some cases, demonstrating the feasibility of the approach proposed. This approach, after further studies, or combined with more measures such as Sample Entropy, is certainly very promising in becoming an early diagnosis tool based solely on body temperature temporal patterns, which is of great interest in the current Covid-19 pandemic scenario.

## 1. Introduction

The current Covid-19 pandemic has demonstrated once more how important it is to closely monitor body temperature at a very large scale, remotely, and continuously. Unfortunately, a suitable scheme that addresses simultaneously all these issues is still unavailable. Only some progress in terms of thermal imaging has been made and is currently in use. However, at a general clinical level, and for disease detection, infra-red single temperature readings are still the standard practice. This is a very poor approach that will have to change in the coming years in order to improve, not only readiness against other possible future pandemics, but also the treatment of any other pathology where body temperature can play an important role in early diagnosis and prognosis [1].

Although the infra-red clinical temperature measuring technology is very convenient in terms of non-obtrusiveness and lack of contact (distance), in its current form it is certainly not more than a plain fever/no fever assessment tool [2]. It still overlooks the richer physiological information yielded by the output of the thermo-regulatory system that can be obtained from a temporal continuum perspective instead, even before fever is present [3]. In fact, the analysis of body temperature time series certainly provides a deeper insight into patient status, as some studies have already shown [4,5]. Moreover, there are portable body temperature monitors available in the market already, certified for medical use, that can be employed in a similar way as classical Holter monitors for long-term high-frequency temperature data logging.

In this respect, the study [1] described how important it is to monitor the body temperature continuously during 24 h. This work also suggested to use both central and peripheral body temperature, and adopt a chronobiological and/or complexity analysis perspective to maximize the clinical information extracted from body temperature time series. In [6], continuous temperature monitoring was applied to patients during 24 h at a sampling rate of 0.1 Hz. This study assessed the differences among patients with sepsis, septic shock and systemic inflammatory response syndrome using Wavelet features mainly. The results confirmed the validity of this approach, with a classification accuracy of up to 80%, able to significantly distinguish among some of such pathologies. The work in [7] used also body temperature time series (1 min sampling rate, several days, critical patients) to demonstrate an association between the mortality rate at an ICU, and the circadian alterations found in core body temperature of the patients. Drewry et al. [8] analyzed 72 h periods of body temperature data (for patients staying at an ICU, with measurements every 3–4 h) in terms of maximum, minimum, and variations, in order to predict sepsis in afebrile patients, and therefore anticipate diagnosis and treatment. The temperature curve analysis proposed was able to achieve an abnormal pattern detection sensitivity of 69%. Anyway, clinical studies based on continuous analysis of temperature data are still scarce and largely outnumbered by studies based on other continuous physiological variables.

Regarding mathematical tools used to analyze temperature time series, entropy measures are clearly becoming one of the most usual tools, probably due to their success on other types of clinical temporal data. For example, Permutation Entropy (PE) [9] and Approximate Entropy (ApEn) [10] were used in [11] to successfully discriminate between continuous temperature records from healthy patients and subjects that developed a fever the day before the records were acquired (but not during monitoring). A classification accuracy of 90% was achieved using the method proposed. Another analysis of temperature data using ApEn [5] was demonstrated to provide significant predictive value about the outcome of critical patients in an ICU, with an accuracy of 70% for distinguishing between patients that survived from those that did not. The study in [12] processed 3 h body temperature records at 0.1 Hz from preterm infants to find a possible association with gestational age and respiratory morbidity using Detrended Fluctuation Analysis (DFA) [13] and Sample Entropy (SampEn) [14]. The DFA results exhibited a significant relationship with patient demographics and respiratory disease severity. Other features, such as SampEn, mean temperature, or coefficient of variation, provided a much more modest association with the status of the infants, if any. Other works, such as [3], have focused their attention on predicting fever peaks before they actually occur. Using logistic regression and linear discrimination analysis models, they were able to predict around 84% of the fever spikes, using features such as ApEn, gradient between core and peripheral temperature, and Cross-ApEn [15], among others (24 h continuous monitoring).

Another line of research using temperature time series that is gaining momentum among the scientific community is to devise new signal processing techniques that enable a diagnosis based on temporal fever patterns (differential diagnosis). This is the topic addressed in the present work. This research is of great interest to provide tools to distinguish among some diseases inexpensively, remotely, and at any place and at any time. It also takes advantage of the recent progress made on the two previous key issues described: the analysis of continuous body temperature time series, and the application of entropy measures [16]. For instance, the work [17] used SampEn to find differences between dengue, tuberculosis, and other non-infectious and non tubercular bacterial infections. The classification accuracy achieved spanned from 0.60 up to 0.77, depending on the diseases under comparison. Other similar studies were devoted to find specific fever patterns in tuberculosis [18], or used a set of multiple features to increase classification accuracy of such patterns [19]. Using a training set, it is also possible to accomplish this task using tools such as neural networks [20]. In a more general way, other works studied fever patterns from a descriptive point of view [21]. This line of research is still a rather unexplored field that certainly deserves more attention.

**Figure 1:**
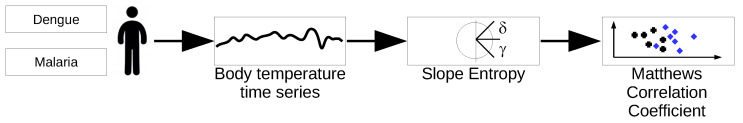
Block diagram of the study. Body temperature records from dengue and malaria patients were recorded over 24 h. The corresponding time series were processed using Slope Entropy, and then classified using a single threshold obtained from the Receiver Operating Characteristic (ROC) Curve. The performance was assessed using the Matthews Correlation Coefficient.

This paper proposes a scheme for assessing the differences between body temperature records coming from a variety of pathological backgrounds, as in [17]. The analysis was based on body temperature records of 24 h, 1 min sampling period, from patients that specifically developed a fever due to malaria or dengue diseases. The analysis was not only focused on the global differences of the entire records, but also on quantifying the differences along or at some temporal windows. This way it would not be necessary to monitor for 24 h before the differences became detectable, and diagnosis could be anticipated several hours, triggering earlier patient isolation and/or treatment, if necessary. The entropy measure employed was Slope Entropy (SlopEn), a recently proposed method [22] based on gradient patterns between consecutive samples. The results achieved demonstrated a high disease classification potential that could be re–assessed using also other pathologies in order to achieve an additional inexpensive and unobtrusive diagnosis tool. A block diagram of the study is shown in Figure 1. Each step in this diagram will be described in detail in the next Sections.

## 2. Materials and Methods

### 2.1. Dataset

The experimental dataset included 31 body temperature time series of dengue (DE) patients (18 males), and 16 of malaria (MA) patients (12 males), diagnosed according to specific clinical findings. Subjects with undifferentiated fever, a febrile illness accompanied by non-specific symptoms, admitted to the hospital and who met the following criteria were included in the study:

1. Fever of more than or equal to 7 days duration.
2. Individuals with an intact tympanic membrane.
3. Subjects aged between 18–65 years.

The Institutional Ethics Committee approval was obtained for the study on 15/01/2014 (IEC KMC MLR 01-14/13). Written informed consent was obtained from each participant after explaining the study in the language they understand. Confidentiality was maintained about their identity.

The time series were acquired once over 24 h, starting at 9:00, with a sample per minute, 1440 temperature readings in total. Before starting the continuous tympanic temperature recording, subjects’ auditory canals were examined and any ear wax, if present, was removed from the auditory canal using normal saline. After the auditory canal was dried, the probe was fixed for recording. A temperature probe was gently inserted into the auditory canal which projected towards the tympanic membrane. Another end of the temperature probe was connected to the temperature monitoring device and the recorded temperature was stored. Prior to temperature recording, the study subjects were informed not to take a shower and not to perform any strenuous exercises. Temperature of the subjects was recorded when the ambient temperature was in the range of 22 to 40 ${}^{\circ }$C.

**Figure 2:**
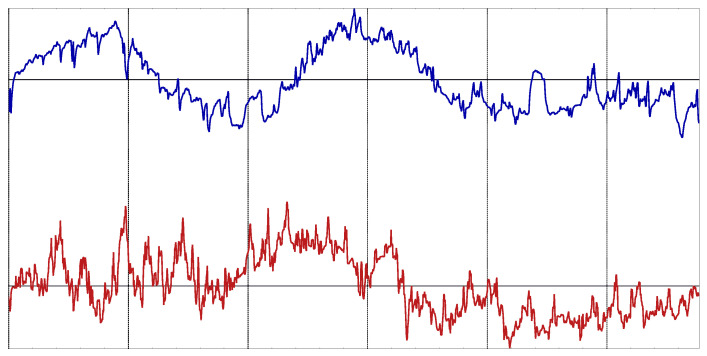
Example of body temperature records from the experimental dataset. Bottom record corresponds to a dengue patient. Top record corresponds to a malaria patient.

All the records were normalized before the classification analysis: zero mean and unit variance. No other preprocessing took place. An example of one record of each class is shown in Figure 2.

These records have not been used in any other prior study yet, although they were acquired in the same clinical setting as the dataset used in [17]. The clinical contributors of this paper from Kasturba Medical College (India) are conducting an ongoing effort of recording body temperature data from a disparity of pathologies: dengue, malaria, tuberculosis, leptospirosis, thyroiditis, enteric fever, pyogenic sepsis, and others, and when a significant number of records of each type becomes available, we study their properties and the best methods to yield a differential diagnosis based solely on time series analysis, as was the case in [17,18,19,20] and in the present paper.

### 2.2. Slope Entropy

SlopEn was first described in [22]. It is based on computing relative frequencies of subsequences, as many other entropy methods, but instead of using amplitude or ordinal information, it uses as symbols the representation of the slope between two consecutive samples.

Given an N–length input time series $x=\left\{x_{0},x_{1},\ldots ,x_{N-1}\right\}$ and m–length subsequences of x, $x_{j}^{m}=\left\{x_{j},x_{j+1},\ldots ,x_{j+m-1}\right\}$, a symbolic pattern is obtained for each $x_{j}^{m}$ by thresholding each difference $d=x_{j+1}-x_{j}$ as:

- Symbol 2, if d>γ.
- Symbol 1, if d≤γ and d>δ.
- Symbol 0, if $\left|d\right|\leq \delta$.
- Symbol −1, if d<−δ and d≥−γ.
- Symbol −2, if d<−γ.

being γ and δ two input parameters, with γ>δ>0.

Once all the symbols are computed for a subsequence, a pattern–matching process takes place in order to update the relative frequency of each one in a list dynamically filled with the patterns found so far. The final vector of relative frequencies is used to compute the result using a Shannon approach [23]. An algorithm for computing SlopEn is shown below (Algorithm 1):
**Algorithm 1** Computing SlopEnf=**SlopEn**(x, *m*, γ, δ)f=0.0Π←⌀      (Empty list of patterns found)**for** j=0,…,N−m    Ω←⌀      (Empty pattern)    **for** i=j+1,…,j+m−1       $d=x_{i+1}-x_{i}$       **if** ($\left|d\right|\leq \delta$)      $\Omega \leftarrow “0^{\prime \prime }$      (Add symbol 0)       **if** (δ<d≤γ)     $\Omega \leftarrow “1^{\prime \prime }$      (Add symbol 1)       **if** (d>γ)          $\Omega \leftarrow “2^{\prime \prime }$      (Add symbol 2)       **if** (−γ≤d<−δ)   $\Omega \leftarrow “-1^{\prime \prime }$      (Add symbol −1)       **if** (d<−γ)        $\Omega \leftarrow “-2^{\prime \prime }$      (Add symbol −2)    Π←Ω$\forall \Omega_{k}\in \Pi$    $p_{k}=\left(\#\left(\Omega =\Omega_{k}\right)\in \Pi \right)/(\#$ unique Ω∈Π)      (Compute relative frequency of each pattern, #=sizeof operator)    $f=f-p_{k}\;*\;$log$\left(p_{k}\right)$

The final values were normalized by the maximum SlopEn obtained in order to keep their range between 0 and 1. A more detailed SlopEn algorithm is described in [22]. The SlopEn result of each record was the single classification feature to be used in the performance analysis carried out in Section 3.

For comparative purposes, SampEn was also computed in the experiments. This measure is one of the most successful methods for time series classification [24,25,26,27], including temperature records [17], and it is therefore a good cornerstone on which to build the conclusions for SlopEn.

### 2.3. Performance Assessment

The Matthews Correlation Coefficient (MCC) [28] was used in the present work in order to quantify the performance of the method described. This is a binary classification performance metric based on considering the Positives (P, dengue in this case) and Negatives (N, malaria) as two different variables (the classification labels for the two classes under analysis), and computing their correlation. The closer the correlation is to 1, the better is the classification. It is initially smaller than others, reaching 0.5 when 75% of the predictions are correct. It is based on the also well known phi-coefficient [29].

The MCC is better suited for imbalanced classes than other methods or variables. It ranges between −1 and +1, with +1 accounting for a perfect classification, 0 for a random classification, and −1 for a complete reverse classification. No class is more important than the other, however unbalanced the partition is. The MCC results are symmetric. In a binary case, this measure can be easily computed as:

$$\mathrm{MCC}=\frac{\mathrm{TP}\times \mathrm{TN}-\mathrm{FP}\times \mathrm{FN}}{\sqrt{\left(\mathrm{TP}+\mathrm{FN})(\mathrm{TP}+\mathrm{FP})(\mathrm{TN}+\mathrm{FP})(\mathrm{TN}+\mathrm{FN}\right)}}$$

with TP = True Positives (actual positives that are correctly labelled as P), FN = False Negatives (actual positives that are incorrectly labelled as N), TN = True Negatives (actual negatives that are correctly labelled as N), and FP = False Positives (actual negatives that are incorrectly labelled as P). For balanced sets, there is a good correspondence between accuracy and MCC [30], whereas for imbalanced cases, accuracy can be overoptimistic. It is then where MCC exhibits its superior robustness and reliability [28]. For a highly truthful classification, MCC should be as close to 1 as possible, at least above 0.5, in general. Scores in the vicinity of 0 account for mediocre classifications (random guess) that should not be considered as good prediction tools. The classification threshold was obtained from the Receiver Operating Characteristic (ROC) Curve in each experiment, as the point on the curve closest to coordinates (0,1), a standard procedure in many similar works [31].

In order to compare the results of the present paper with previous studies that did not use MCC but the classification accuracy instead, in some cases the results were also quantified in terms of sensitivity (true positive rate, correctly classified dengue patients), specificity (true negative rate, correctly classified malaria patients), and accuracy (ratio or percentage of total records correctly classified).

## 3. Experiments And Results

### 3.1. Input Parameter Configuration

**Figure 3:**
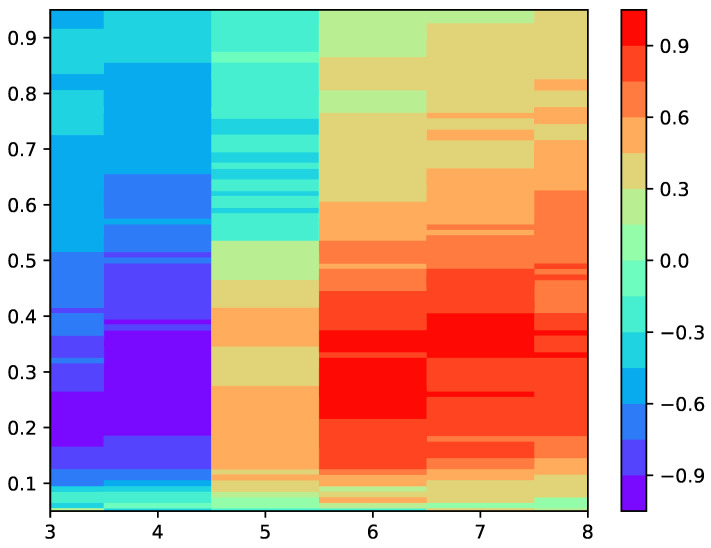
Heat map featuring the values of MCC achieved for different *m* and γ values of SlopEn. δ was kept constant at $1\times 10^{-6}$. The region of highest performance in terms of MCC lay in the region *m* = 6, 7, and γ=[0.25,0,35]. There was a reverse region in the vicinity of m=4 and similar γ values. Except for m=5 and high γ values, the performance seemed to be high and stable. Instead of using the peak parameter values (m=6,γ=0.27, MCC =0.9529, only two points with this performance), a more centred and stable region was selected (m=6,γ=0.25, MCC =0.9066, with 24 points with this performance in the vicinity of that specific parameter configuration) for the experiments.

The first step of the experiments was to find a suitable input parameter configuration for SlopeEn and the two diseases under analysis, dengue and malaria. This search was confined to parameters *m* and γ, with $\delta =1\times 10^{-6}$ in all cases. The results of this preliminary step are shown as a heat map in Figure 3.

It can be stated that the classification performance in terms of MCC was reasonably quite stable, with almost the entire grid above 0.5 (or its reverse), and many regions around 0.8–0.9, making the selection of the input parameter values less critical. For stability, we chose m=6 and γ=0.25.

### 3.2. Classification Performance

**Figure 4:**
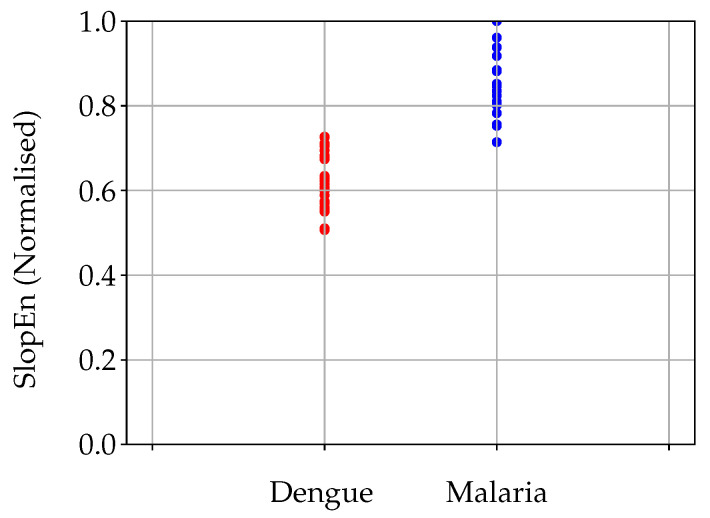
Cloud plot of the two classes under analysis, dengue and malaria. Dengue records had a significant lower SlopEn values than malaria records. The separability of these two types of temperature records is visually very apparent, as confirmed by the numerical result of MCC (0.9066) for m=6 and γ=0.25.

With the specific parameter configuration stated in the previous section, the MCC value obtained was 0.9066 (98 % of time series correctly classified, a sensitivity of 1, and a specificity of 0.93), in other words, DE and MA records were clearly separable using SlopEn. This discriminant power is illustrated in Figure 4.

Using SampEn, the highest performance was achieved using m=3,4, and r= 0.25–0.50, yielding MCC = 0.6347, sensitivity = 0.81 and specificity = 0.87. This was also a good performance that confirms the suitability of SampEn to this task, as demonstrated in [17].

A Leave One Out (LOO) cross validation analysis was also carried out for a more robust demonstration of the separability of the two classes, DE and MA. Since the number of instances of each class was not very big, this method was considered better suited to this case [32]. This analysis was based on removing one time record from each class, computing a classification threshold from the remaining records using their ROC curve, and then applying the threshold obtained to the initially removed records for their classification. This process was repeated until all the time records were left-out once in the majority class, DE (imbalanced dataset), although some instances of the minority class might have been used more than once [33] due to random oversampling [34]. This LOO analysis yielded the following results: 94% of the time series correctly classified, with a standard deviation of 0.0185. As usual, the performance with LOO was worse than using the entire dataset for classification (98% vs. 94% correctly classified instances), but still very high.

### 3.3. Window Analysis

From a prompt diagnosis perspective, and taking into account that fever frequently undergoes chronobiological variations, it would be convenient to assess the separability between the temperature records again, but using shorter time windows. This way, it will not necessary to wait 24 h, and it could be hypothesized that intervals of greater differences could be identified for better temperature time series characterization.

**Figure 5:**
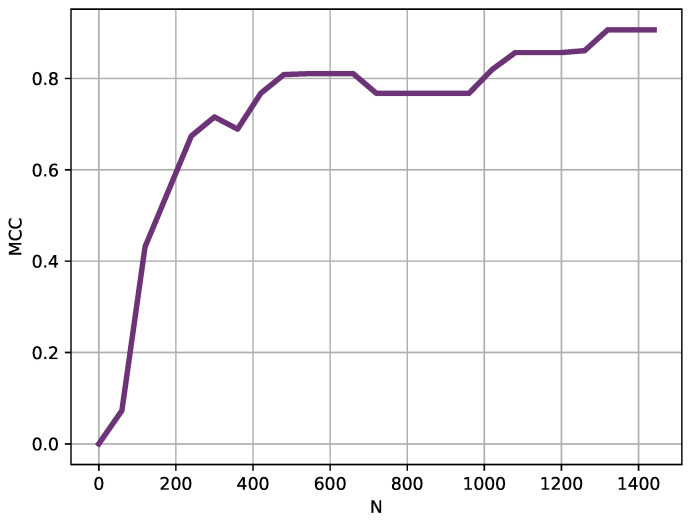
Length (*N*) analysis results. Instead of using the entire records, shorter versions were used in this analysis. Starting at 60 samples long, length was progressively increased in 60 samples steps. With lengths slightly above 200 samples, the MCC obtained was very high already, that is, the records were clearly separable using only 3–4 h of temperature data.

The experiments were therefore repeated using the same parameter configuration, using increasing lengths in 1 h steps (60 samples), as depicted in Figure 5.

This last experiment offered a clear but incomplete picture of the possible effect of temporal windows. In order to get the full picture, it was also necessary to assess the effect of a sliding window of fixed size instead, related to possible chronobiological changes in the dynamics of the temperature data of each disease, some of which have already been reported in the scientific literature [21,35].

**Figure 6:**
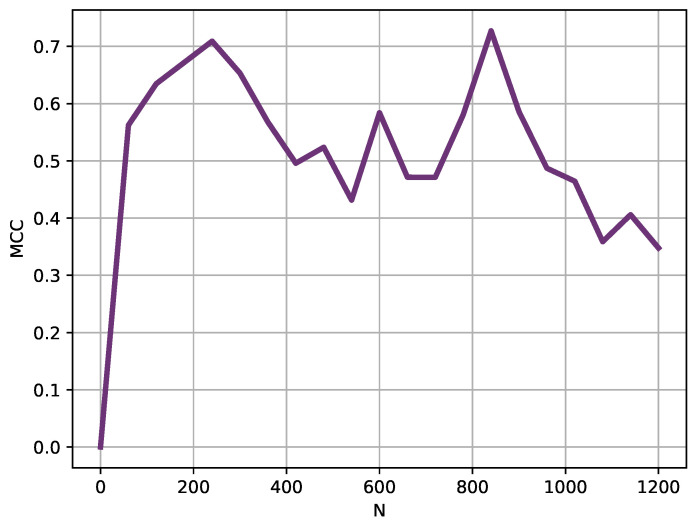
Sliding window analysis results. Using a moving window of 240 samples, this analysis shows the MCC achieved depending on the location of such window at 60 samples steps. Two performance peaks become apparent at locations close to sample 200 and sample 800. It could be hypothesized that fever patterns are not equally different at any point in a 24 h cycle, and there are regions where differences become more apparent.

Using a sliding window of 240 samples (4 h), for which MCC achieved a high MCC value of at least 0.70 (Figure 5), the classification experiment was repeated moving the starting point in 60 samples steps. These results are depicted in Figure 6.

### 3.4. Generalization Analysis

The comparison between DE and MA temperature records has provided clear evidence of the possible separability of both diseases based solely on the SlopEn analysis of the corresponding time series. However, it could be stated that these two diseases are not more than a successful example that does not necessarily demonstrate any generalization capability when applied to other diseases.

In order to avoid this possible overfitting or misinterpretation, the experiments in this subsection were devised to explore the behaviour of the method when used with fever time series coming from other diseases. It is important to note, though, that there are not publicly available body temperature continuous time series databases (as far as we know), the additional data used here were also acquired in the same clinical setting as the main experimental database, and these experiments were not as far-reaching as for the initial database.

The first additional disease studied was leptospirosis (LE), since we already had 15 records of this type. The separability analysis between leptospirosis and dengue was carried out in the same way, with a parameter optimization stage based on MCC computation. The highest performance was achieved at region *m* = 3, 4, and γ= 0.25–0.50, with MCC = 0.8500 (sensitivity = 1, specificity = 0.87), quite stable, with half the parameter space above 0.60 at least (sensitivity and specificity above 0.80). The MCC result achieved using the same parameter configuration as for DE and MA was 0.6604. The results obtained with SampEn for LE and DE were sensitivity = 0.90, specificity = 0.86, and MCC = 0.7163, using other input parameter values specifically optimized for this method.

In the case of leptospirosis and malaria, the performance achieved was slightly lower but still very significant. In the parameter region of m=5,6 and γ>0.6, MCC ≥0.5557 (sensitivity = 0.73, specificity = 0.85), with a maximum of 0.6310. The MCC result achieved using the same parameter configuration as for DE and MA was 0.2996 (sensitivity = 0.8, specificity = 0.56). Therefore in this case this configuration should be replaced by a more suitable one as described before. SampEn failed in this case to find significant differences, with best MCC = 0.3133, sensitivity = 0.80, and specificity = 0.56, clearly imbalanced, whatever were the parameter values employed.

**Figure 7:**
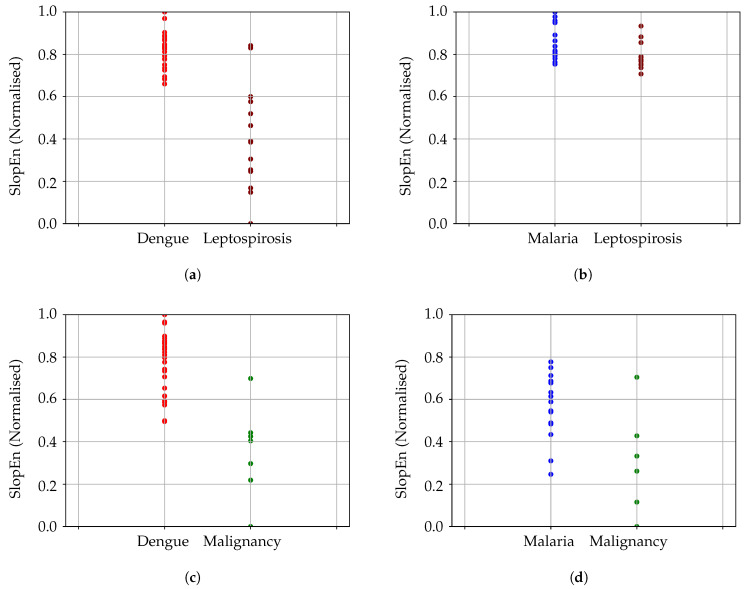
Comparison between pairs of diseases: dengue, malaria, malignancy, and leptospirosis. It has to be noted that the above results were achieved using different input parameter configurations. (**a**) Comparison of dengue and leptospirosis results. (**b**) Comparison of malaria and leptospirosis results. In this case, the performance is lower. In addition, the points are very close, which makes it difficult to see the possible separability, despite the numerical results in this regard. (**c**) Comparison of dengue and malignancy results. (**d**) Comparison of malaria and malignancy results. As in Figure 7b, the performance is lower and more than a single point falls almost in the same spot, giving the impression the classification error is higher, despite having only two incorrectly classified records for malaria, and 1 for malignancy.

We also used seven records of body temperature corresponding to malignancy (ML). When compared to dengue, the MCC achieved was greater than 0.82, for m=3,4 and γ= 0.70–0.80, sensitivity = 1, specificity = 0.85. For m=6 and γ=0.25, MCC was 0.4814 (sensitivity = 0.77, specificity = 0.85). The highest performance for SampEn was sensitivity = 0.83, specificity = 0.85, with MCC = 0.5534. Comparing malignancy and malaria, the MCC results were 0.5635 for m=3 and γ>0.7 (sensitivity = 0.81, specificity = 0.85), with MCC = 0.2441 using the initial parameter configuration. Again, it would be better to choose a more optimal input parameter configuration in this case. SampEn achieved an MCC = 0.4377, with a sensitivity of 0.75, and a specificity of 0.85. All these results using only seven ML records should be taken with caution due to the low number of records, but they were included since at least a general trend could still be observed. The results of all these experiments are depicted in Figure 7.

In another group of experiments, the same database of body temperature records as in [17] was used, with additional diseases: tuberculosis, fevers of non-tubercular origin, and fevers without evidence of infection (dengue was not used again). In that case, Sample Entropy (SampEn) was the entropy measure applied, and the records were also low–pass filtered. Trace segmentation [36], used as an additional feature selection method in [17], was not used here. Further details of these datasets can be found in the original paper.

Some of those additional diseases were analyzed for separability using SlopeEn. They were not compared with the present dataset of MA or DE since they were not filtered as in [36]. For comparative purposes with results from that previous study, sensitivity and specificity were the important metrics in these cases.

For non-tubercular and tuberculosis fevers, the maximum MCC obtained was 0.6849, when m=4, and almost for any γ. This was a relatively low performance value, but in the original paper it was even lower, since this was a difficult classification case. In [17], the maximum sensitivity and specificity were 0.61 and 0.68 respectively (MCC was not used there), whereas in the present case were 0.75 and 0.61, or 0.68, 0.67, depending on the specific parameter configuration, higher than using SampEn anyway.

When comparing non-infectious with tuberculosis records, the MCC achieved using SlopEn was 0.6607, with 0.61 and 0.71 for sensitivity and specificity (with *m* = 6, 7, and for almost any γ). In this case the performance using SampEn was higher, with 0.78 for the sensitivity, and 0.75 for the specificity.

Finally, the analysis using non-infectious and non-tubercular records yielded non-significant results for the MCC based on SlopEn (around 0.05, for specificities and sensitivities in the vicinity of 0.50). However, SampEn was able to find differences, with 0.64 sensitivity, and 0.75 specificity [17].

### 3.5. Results Summary

The results of all the previous classification experiments described in this Section are summarized in Table 1. The method, SlopEn or SampEn, that achieved the highest performance, is highlighted.

## 4. Discussion

Finding a method to quantify the differences of body temperature curves among diseases can be a first step towards a new inexpensive diagnosing tool that fulfils the current and future needs of mass-analysis, patient isolation, and continuous and long-term monitoring. Recent efforts have been devoted successfully to accomplish this goal [17,18,19,20], although this is still a research field in its infancy.

Specifically, the present study is mainly based on [17]. However, as [17] employed SampEn as the discriminating feature, when SlopEn was not available yet, and since SlopEn seemed very promising as it outperformed SampEn in many comparative analyzes [22], it was necessary to assess the capability of this new method in the framework of temperature time series. The present study used a more diverse experimental dataset, implemented a novel windowing approach, analyzed the influence of record duration, and did not use any feature extraction method such as Trace Segmentation [17]. Using or combining other approaches based on more features or more sophisticated classification schemes could arguably improve the accuracy achieved, but we leave that approach for future studies.

The first step was to find a suitable input parameter configuration using a grid search for parameters *m* and γ, keeping δ constant. The results are shown in Figure 3. It is important to note that this optimization was not really necessary, since almost all combinations yielded an MCC higher than 0.5, and only a very narrow region for γ<0.15, γ>0.85 or some areas of m=5, yielded MCC results below $\left|0.5\right|$. However, as a general step, this optimization is advisable in every case until there are some recommendations or guidelines in this regard, as there are for other methods more widely characterized [37,38,39,40,41].

The segmentation between DE and MA records was very successful, with a percentage of correctly classified instances of 98%. This performance was confirmed with the LOO analysis, with an averaged performance of 94%. The window analysis provided support to the hypothesis that differences between pathologies can become apparent in a relatively short term, and that those differences can not be uniformly distributed across the time series. Obviously, these temporal variations should be studied on a pathology by pathology basis, but it can arguably be concluded that DE and MA records can be robustly segmented using the method proposed and only 3–4 h of data.

For example, 4 h window at 800 min appears to yield high accuracy. It corresponds to time between 10 pm and 2 am. This corresponds to sleep time and might reduce other interfering factors like physical activity and autonomic changes. It is likely to represent the true basal state of the temperature pattern. It would be logistically convenient to record night temperature patterns. Even blood pressure monitoring, nocturnal blood pressure patterns, are important compared to day time readings.

Unfortunately, there are not many body temperature time series representative of a wide set of pathologies available for a more ambitious study, but it was of paramount importance to demonstrate that the results of DE-MA were not a kind of experiment of one. One of these few additional time series corresponded to 15 patients with leptospirosis, and seven with malignancy, recently acquired by the clinical coauthors of this paper from Kasturba Medical College.

The analysis with the leptospirosis records involved a comparison with DE and MA on a pair-by-pair basis, as done with DE-MA. With LE and DE, the performance was still very high, although the maximum was achieved at a different input parameter configuration. For LE and MA, the performance decreased but still above what can be considered an acceptable performance threshold in MCC, 0.5.

Although seven records do not usually suffice for a statistically significant analysis, we thought the comparison in broader terms would still be worth. In principle, the results followed the same pattern as for the previous diseases, with MCC > 0.82 for ML and DE, and MCC = 0.5635 for ML and MA, not so high. It will be necessary to acquire more records of this kind in order to confirm in future studies if this pathology is so easily distinguishable, but MCC is more robust than plain accuracy and it is reasonable to assume the trend will be the same with a greater number of time series.

The rest of the records used for classification, taken from the [17] study, were a different temperature time series since they were low-pass filtered. The results of the experiments using non-tubercular, tuberculosis, and non-infectious records were not as good as in the previous cases, but still very significant, except for non-infectious and non-tubercular records, the only case where SlopEn was unable to find differences. Others records and combinations in [17] were not addressed in the present paper because, as in the reference study, neither SlopEn nor SampEn were able to find any difference. These records will probably require additional features (average temperature, number of peaks), for a successful classification, or a combination of more methods, as in [42]. Diagnostic accuracy will increase further when clinical details and basic laboratory data are also included in the analysis.

Not all the records were analyzed in the same depth, since that is not possible in a single paper, but of the seven pairs assessed, SlopEn exhibited a high discriminating power in seven. Moreover, SlopEn outperformed SampEn in six pairs. Taking into account that SampEn already exhibited a very high performance in [17] outperforming itself other entropy methods, SlopEn can probably be considered a good choice for this kind of analysis. Besides, there is an input parameter that was not optimized at all, δ, and also the symmetry of the SlopEn thresholds, which could have contributed to a better disease detection. However, a balance between method customization and possible overfitting should be kept.

## 5. Conclusions

SlopEn can be a promising tool for assessing differences among fever patterns in body temperature time series. Frequently, a fever symptom entails additional clinical and lab tests to find out the specific disease causing the fever. These tests can be expensive, time consuming, or not available in many contexts (lack of enough resources, remote or rural locations, ambulatory monitoring). Therefore, a prompt and inexpensive diagnosis would be of great help.

In order to achieve a very robust tool to be applied in real clinical scenarios, further studies will be necessary, adding more temperature time series features, and more entropy related methods that look into amplitude or ordinal patterns, in a complementary way. In addition, more records of each disease, and more diseases, should be included in the experimental set. We are currently addressing this data gap, but it entails a huge effort, and it would be great if other researchers also acquired and shared temperature records to speed up the number of studies related to body temperature with a long term perspective.

Of extraordinary interest are Covid-19 temperature records, but they are currently too difficult and risky to acquire by the clinical staff, specially in overwhelmed healthcare systems. The approach introduced in this and previous papers could be probably be applied not only to patients that have developed the disease, but also to those in a sub-clinical stage, pre and post disease, in order to gain a deeper insight into the evolution of the disease. Another objective of the present paper is precisely to lay the foundations for the analysis of any body temperature record and obtain actionable data for an earlier diagnosis, treatment, or patient isolation if required.

Temperature pattern is likely facilitating the diagnostic work–up by enhancing the pretest probability in one direction. Final diagnosis for therapeutic purposes will be decided by specific tests. Hence, even a moderate degree of accuracy is sufficient for treating physicians in deciding the specific tests instead of ordering broad range of multiple tests. Accuracy will further improve as more and more data sets are collected which will include many more disease conditions.

In summary, despite the possible limitations in terms of number of features, number of signals or number of diseases, this preliminary work provides sound evidence that the analysis of body temperature time series has a huge potential as an inexpensive and unobtrusive diagnosis tool, since SlopEn, and also other methods such as SampEn, have the potential to find differences among records from a varied and diverse set of diseases. The only thing needed is a change in the way body temperature is monitored, from manual isolated readings to continuous high-frequency long-term wireless digital data.

## Figures and Tables

**Table 1:** Summary of the results obtained in all the classification experiments.

Diseases	Method	Sensitivity	Specificity	MCC
(DE, MA)	**SlopEn**	1	0.93	0.9066
	SampEn	0.81	0.87	0.6347
(DE, LE)	**SlopEn**	1	0.87	0.8500
	SampEn	0.90	0.86	0.7163
(MA, LE)	**SlopEn**	0.85	0.73	0.6310
	SampEn	0.80	0.56	0.3133
(DE, ML)	**SlopEn**	1	0.85	0.82
	SampEn	0.83	0.85	0.5534
(MA, ML)	**SlopEn**	0.81	0.85	0.5635
	SampEn	0.75	0.85	0.4377
(NT, TU)	**SlopEn**	0.68	0.67	0.6849
	SampEn	0.61	0.68	–
(NI, TU)	SlopEn	0.61	0.71	0.6607
	**SampEn**	0.78	0.75	–
(NI, NT)	SlopEn	0.55	0.54	0.05
	**SampEn**	0.64	0.75	–

## References

[1] Varela-Entrecanales M., Cuesta-Frau D., Madrid J.A., Churruca J., Miró-Martínez P., Ruiz R., Martinez C. (2009). Holter monitoring of central and peripheral temperature: Possible uses and feasibility study in outpatient settings. J. Clin. Monit. Comput..

[2] Cuesta-Frau D., Varela-Entrecanales M., Valor-Perez R., Vargas B. (2015). Development of a Novel Scheme for Long-Term Body Temperature Monitoring: A Review of Benefits and Applications. J. Med. Syst..

[3] Jordán-Núnez J., Miró-Martínez P., Vargas B., Varela-Entrecanales M., Cuesta-Frau D. (2017). Statistical models for fever forecasting based on advanced body temperature monitoring. J. Crit. Care.

[4] Papaioannou V.E., Chouvarda I.G., Maglaveras N.K., Baltopoulos G.I., Pneumatikos I.A. (2013). Temperature multiscale entropy analysis: A promising marker for early prediction of mortality in septic patients. Physiol. Meas..

[5] Cuesta-Frau D., Varela M., Miró-Martínez P., Galdós P., Abásolo D., Hornero R., Aboy M. (2007). Predicting survival in critical patients by use of body temperature regularity measurement based on approximate entropy. Med. Biol. Eng. Comput..

[6] Papaioannou V., Chouvarda I., Maglaveras N., Pneumatikos I. (2012). Temperature variability analysis using wavelets and multiscale entropy in patients with systemic inflammatory response syndrome, sepsis, and septic shock. Crit. Care (London, UK).

[7] Culver A., Coiffard B., Antonini F., Duclos G., Hammad E., Vigne C., Mege J.L., Baumstarck K., Boucekine M., Zieleskiewicz L. (2020). Circadian disruption of core body temperature in trauma patients: A single-center retrospective observational study. J. Intensive Care.

[8] Drewry A.M., Fuller B., Bailey T., Hotchkiss R.S. (2013). Body temperature patterns as a predictor of hospital-acquired sepsis in afebrile adult intensive care unit patients: A case-control study. Crit. Care (London, UK).

[9] Bandt C., Pompe B. (2002). Permutation Entropy: A Natural Complexity Measure for Time Series. Phys. Rev. Lett..

[10] Pincus S., Gladstone I., Ehrenkranz R. (1991). A regularity statistic for medical data analysis. J. Clin. Monit. Comput..

[11] Cuesta-Frau D., Molina-Picó A., Vargas B., González P. (2019). Permutation Entropy: Enhancing Discriminating Power by Using Relative Frequencies Vector of Ordinal Patterns Instead of Their Shannon Entropy. Entropy.

[12] Jost K., Pramana I., Delgado-Eckert E., Kumar N., Datta A., Frey U., Schulzke S. (2017). Dynamics and complexity of body temperature in preterm infants nursed in incubators. PLoS ONE.

[13] Iyengar N., Peng C.K., Morin R., Goldberger A.L., Lipsitz L.A. (1996). Age-related alterations in the fractal scaling of cardiac interbeat interval dynamics. Am. J. Physiol. Regul. Integr. Comp. Physiol..

[14] Richman J.S., Moorman J.R. (2000). Physiological time-series analysis using approximate entropy and sample entropy. Am. J. Physiol. Heart Circ. Physiol..

[15] Pincus S. (2001). Assessing serial irregularity and its implications for health. Ann. N. Y. Acad. Sci..

[16] Vargas B., Cuesta-Frau D., Ruiz-Esteban R., Cirugeda E., Varela M. (2015). What Can Biosignal Entropy Tell Us About Health and Disease? Applications in Some Clinical Fields. Nonlinear Dyn. Psychol. Life Sci..

[17] Cuesta-Frau D., Miró-Martínez P., Oltra-Crespo S., Molina-Picó A., Dakappa P.H., Mahabala C., Vargas B., González P. (2020). Classification of fever patterns using a single extracted entropy feature: A feasibility study based on Sample Entropy. Math. Biosci. Eng..

[18] Dakappa P.H., Rao S.B., Bhat G.K., Mahabala C. (2019). Unique temperature patterns in 24-h continuous tympanic temperature in tuberculosis. Trop. Dr..

[19] Dakappa P.H., Prasad K., Rao S.B., Bolumbu G., Bhat G.K., Mahabala C. (2017). A Predictive Model to Classify Undifferentiated Fever Cases Based on Twenty-Four-Hour Continuous Tympanic Temperature Recording. J. Healthc. Eng..

[20] Dakappa P.H., Prasad K., Rao S.B., Bolumbu G., Bhat G.K., Mahabala C. (2018). Classification of Infectious and Noninfectious Diseases Using Artificial Neural Networks from 24-h Continuous Tympanic Temperature Data of Patients with Undifferentiated Fever. Crit. Rev. Biomed. Eng..

[21] Ogoina D. (2011). Fever, fever patterns and diseases called ‘fever’—A review. J. Infect. Public Health.

[22] Cuesta-Frau D. (2019). Slope Entropy: A New Time Series Complexity Estimator Based on Both Symbolic Patterns and Amplitude Information. Entropy.

[23] Shannon C.E., Weaver W. (1949). The Mathematical Theory of Communication.

[24] Abásolo D., Hornero R., Espino P., Álvarez D., Poza J. (2006). Entropy analysis of the EEG background activity in Alzheimer’s disease patients. Physiol. Meas..

[25] Sokunbi M.O. (2014). Sample entropy reveals high discriminative power between young and elderly adults in short fMRI data sets. Front. Neuroinform..

[26] Li H., Peng C., Ye D. (2015). A study of sleep staging based on a sample entropy analysis of electroencephalogram. Bio-Med. Mater. Eng..

[27] Cuesta-Frau D., Novák D., Burda V., Molina-Picó A., Vargas B., Mraz M., Kavalkova P., Benes M., Haluzik M. (2018). Characterization of Artifact Influence on the Classification of Glucose Time Series Using Sample Entropy Statistics. Entropy.

[28] Boughorbel S., Jarray F., El-Anbari M. (2017). Optimal classifier for imbalanced data using Matthews Correlation Coefficient metric. PLoS ONE.

[29] Guilford J.P. (1954). Psychometric Methods.

[30] Chicco D., Jurman G. (2020). The advantages of the Matthews correlation coefficient (MCC) over F1 score and accuracy in binary classification evaluation. BMC Genom..

[31] Song B., Zhang G., Zhu W., Liang Z. (2013). ROC operating point selection for classification of imbalanced data with application to computer-aided polyp detection in CT colonography. Int. J. Comput. Assist. Radiol. Surg..

[32] Wong T.T. (2015). Performance evaluation of classification algorithms by k-fold and leave-one-out cross validation. Pattern Recognit..

[33] Weiss G. (2004). Mining with rarity: A unifying framework. SIGKDD Explor..

[34] Kasem A., Ghaibeh A., Moriguchi H. (2016). Empirical Study of Sampling Methods for Classification in Imbalanced Clinical Datasets. International Conference on Computational Intelligence in Information Systems.

[35] Romanovsky A., Simons C., Kulchitsky V. (1998). “Biphasic” fevers often consist of more than two phases. Am. J. Physiol..

[36] Cuesta-Frau D., Pérez-Cortes J.C., García G.A. (2003). Clustering of electrocardiograph signals in computer-aided Holter analysis. Comput. Methods Programs Biomed..

[37] Cuesta-Frau D., Murillo-Escobar J.P., Orrego D.A., Delgado-Trejos E. (2019). Embedded Dimension and Time Series Length. Practical Influence on Permutation Entropy and Its Applications. Entropy.

[38] Alcaraz R., Abásolo D., Hornero R., Rieta J. Study of Sample Entropy ideal computational parameters in the estimation of atrial fibrillation organization from the ECG. Proceedings of the 2010 Computing in Cardiology.

[39] Aboy M., Cuesta–Frau D., Austin D., Micó–Tormos P. Characterization of Sample Entropy in the Context of Biomedical Signal Analysis. Proceedings of the 2007 29th Annual International Conference of the IEEE Engineering in Medicine and Biology Society.

[40] Alcaraz R., Abásolo D., Hornero R., Rieta J.J. (2010). Optimal parameters study for Sample Entropy-based atrial fibrillation organization analysis. Comput. Methods Programs Biomed..

[41] Yentes J.M., Hunt N., Schmid K.K., Kaipust J.P., McGrath D., Stergiou N. (2013). The Appropriate Use of Approximate Entropy and Sample Entropy with Short Data Sets. Ann. Biomed. Eng..

[42] Cuesta-Frau D., Miró-Martínez P., Oltra-Crespo S., Jordán-Núñez J., Vargas B., González P., Varela-Entrecanales M. (2018). Model Selection for Body Temperature Signal Classification Using Both Amplitude and Ordinality-Based Entropy Measures. Entropy.

